# Antibiotic Prescribing Patterns and Guideline Concordance for Uncomplicated Urinary Tract Infections Among Adult Women in the US Military Health System

**DOI:** 10.1001/jamanetworkopen.2022.25730

**Published:** 2022-08-04

**Authors:** Jacqueline Y. Kikuchi, Amanda Banaag, Tracey P. Koehlmoos

**Affiliations:** 1 Department of Gynecology and Obstetrics, Johns Hopkins University School of Medicine, Baltimore, Maryland; 2 Department of Preventative Medicine and Biostatistics, Uniformed Services University, Bethesda, Maryland; 3 Henry M. Jackson Foundation for the Advancement of Military Medicine, Inc, Bethesda, Maryland

## Abstract

**Importance:**

Urinary tract infections (UTIs) are one of the most commonly diagnosed infections, and prior studies have reported discordance in antibiotic treatment with the Infectious Diseases Society of America (IDSA) guidelines.

**Objective:**

To assess IDSA guideline concordance rates for women with uncomplicated UTIs treated with antibiotics, and compare concordance rates between different specialty field.

**Design, Setting, and Participants:**

Retrospective cross-sectional study of health care claims data from the US Military Health System Data Repository, which contains comprehensive health care encounter and claims data for all military beneficiaries. Participants were adult women between the ages of 18 to 50 years with uncomplicated UTIs from October 1, 2017, to September 30, 2019. Data extraction and analysis were performed in 2022. Patients with diagnosis of UTI in the preceding 6 months, current pregnancy, history of pyelonephritis, history of diabetes, history of organ transplant, history of human immunodeficiency virus, immunosuppression, renal insufficiency, urinary tract abnormalities, or history of urologic procedures were excluded.

**Exposures:**

Antibiotic treatment for uncomplicated UTIs. Only antibiotics received within 1 day after the diagnosis were analyzed. The IDSA recommends the following antibiotics as first-line therapy: nitrofurantoin, trimethoprim-sulfamethoxazole, fosfomycin, pivmecillinam.

**Main Outcomes and Measures:**

The IDSA guideline concordance rates were calculated as the number of patients receiving first-line antibiotic therapy divided by the total number of cases for uncomplicated UTIs.

**Results:**

A total of 46 793 adult women (67.3% [31 475 of 46 793] aged 18-34 years; 38.2% [31 475 of 46 793] of White race) were diagnosed with uncomplicated UTIs with 91.0% receiving guideline-concordant antibiotic treatment. In comparison with obstetrics and gynecology, IDSA guideline-concordant treatment was more likely in internal medicine (adjusted odds ratio [aOR], 2.87; 95% CI, 2.73-3.03), family medicine (aOR, 1.81; 95% CI, 1.76-1.87), surgery (aOR, 1.51; 95% CI, 1.36-1.67), and emergency medicine (aOR, 1.36; 95% CI, 1.32-1.39) and less likely in urology (aOR, 0.40; 95% CI, 0.38-0.43). Compared with direct military care, private sector care had lower concordance rates (aOR, 0.63; 95% CI, 0.62-0.64).

**Conclusions and Relevance:**

In this cross-sectional study of antibiotic treatments for uncomplicated UTIs in a universally insured population, the IDSA guideline-concordance rate was high at 91.0% with higher rates in direct military care compared with private sector care. There were higher rates in general medical specialties, surgery, and emergency medicine and lower rates in urology and obstetrics and gynecology. These results further enhance the literature on current antibiotic prescribing practices for uncomplicated UTIs in adult women.

## Key Points

**Question:**

What are the Infectious Diseases Society of America (IDSA) guideline concordance rates in the treatment of uncomplicated acute cystitis with antibiotics, and are there differences in IDSA guideline-concordance rates among various specialties?

**Findings:**

In this cross-sectional study of 46 793 adult women in the US Military Health System, the IDSA guideline-concordance rate was 91%. The specialty fields of internal medicine, family medicine, primary care, surgery, and emergency medicine had higher IDSA concordance rates; urology as well as obstetrics and gynecology had lower IDSA concordance rates.

**Meaning:**

In this study, IDSA concordance rates for uncomplicated cystitis were found to be higher than what has been previously reported with substantial variation among different specialties.

## Teaser

This cross-sectional study assesses the Infectious Diseases Society of America guideline-concordance rate for adult women with uncomplicated urinary tract infections treated with antibiotics in the US military health system.

## Introduction

Urinary tract infections (UTIs) are the most commonly diagnosed outpatient infection. In the US, more than 7 million people are referred to physicians for UTIs each year,^1,2^ and UTIs account for approximately 15% of all antibiotic prescriptions^3,4^ with an annual cost to the US health care system of approximately $1.6 billion.^5,6,7^

In 2011, the Infectious Diseases Society of America (IDSA) updated their international clinical practice guidelines for the treatment of acute uncomplicated cystitis in premenopausal women due to the increasing rate of antimicrobial resistance.^8^ For the optimal treatment of uncomplicated cystitis, the IDSA recommends nitrofurantoin monohydrate/macrocrystals, trimethoprim-sulfamethoxazole, fosfomycin trometamol, and pivmecillinam as first-line therapy. Despite clear IDSA guidelines, practice patterns vary widely with numerous studies showing substantial discrepancies between clinical practice guidelines and antibiotic prescribing practices.^1,9,10,11,12^ A recent study^9^ reported that the rate of IDSA antibiotic guideline concordance (hereinafter, guideline concordance) for UTIs ranged from 58.4% to 64.6%, and several studies^2,10,13,14,15^ have shown that obstetricians and gynecologists are more likely to prescribe a first-line antibiotic compared with other clinicians in other specialties.

Antibiotic treatments for UTIs have been evaluated in a variety of different health care settings,^1,9,10,11,12^ but, to our knowledge, treatment patterns have not yet been studied in the Military Health System (MHS). The MHS provides health care to 9.6 million beneficiaries including active-duty personnel, retirees, and their dependents through the Department of Defense health insurance known as TRICARE.^16^ The MHS delivers care in 2 distinct settings: direct care through more than 350 military health facilities and via the US private sector care through civilian fee-for-service facilities. The ability to compare direct care and private sector care is a unique aspect of the MHS. Evaluating antibiotic prescribing patterns for uncomplicated UTIs in this universally insured population may help provide valuable insights on antibiotic treatment patterns in an optimal care environment. In addition, the Department of Defense formally chartered an antibiotic stewardship program in 2017, which has been implemented across the MHS; however, currently there is no specific requirement that hospitals have a specific outpatient or UTI protocol. Thus, the existence of any of these types of antibiotic stewardship programs varies across the MHS.

The primary objective of this study was to assess the IDSA guideline-concordance rate for adult women with uncomplicated UTIs in the MHS treated with antibiotics. Secondary objectives were to evaluate differences in IDSA guideline concordance rates between different clinician categories (physicians, physicians assistants and nurse practitioners, and other), specialties (general clinician, family medicine, internal medicine, primary care, emergency medicine, surgery, obstetrics and gynecology, urology, and other), and care settings (direct care and private sector care), and assess antibiotic overtreatment rates for uncomplicated UTIs.

## Methods

### Study Design and Population

This retrospective cross-sectional study used health care claims data from the MHS Data Repository (MDR) of all female TRICARE beneficiaries aged 18 to 50 years with uncomplicated UTIs from October 1, 2017, to September 30, 2019. This study was conducted in accordance with the Strengthening the Reporting of Observational Studies in Epidemiology (STROBE) reporting guideline. This study was reviewed and found exempt by the Uniformed Services University of the Health Sciences Institutional Review Board. Informed consent of study participants was not applicable given the use of secondary encounter and claims data. The MHS provides worldwide health care and coverage to approximately 9.6 million TRICARE beneficiaries, with 15% comprising active-duty personnel and the remaining 85% comprising dependent family members and retirees.^16^ Approximately 50% of MHS beneficiaries are women, thereby composing one of the largest cohorts of women in the US under a single health care system.^17,18^ The beneficiary population is considered to be demographically representative of the adult US population from age 18 to 64 years.^19,20,21^ The MDR contains comprehensive health care encounter and claims data for all MHS beneficiaries. It does not include care received under the Veterans Health Administration.

Uncomplicated UTIs were defined using the *International Statistical Classification of Diseases and Related Health Problems, Tenth Revision (ICD-10)* code of N39.0. To exclude patients with recurrent UTIs, only initial incidences of uncomplicated UTI without a history of UTI in the 6 months prior were included in the analysis. Antibiotics prescribed in association with the *ICD-10* diagnosis code of N39.0 were extracted, and only antibiotics prescribed within 1 day after the diagnosis were used in the analysis. IDSA guideline concordance was defined as an antibiotic prescription of either nitrofurantoin monohydrate/macrocrystals, trimethoprim-sulfamethoxazole, fosfomycin trometamol, or pivmecillinam.^8^ Antibiotic dosage and duration data were not collected. Antibiotic overprescribing was defined as prescribing more than 1 antibiotic in association with the *ICD-10* diagnosis code N39.0. The full list of antibiotics is available in eTable 1 in the Supplement. Data on any laboratory orders in association with the *ICD-10* code of N39.0 were not collected.

The IDSA guidelines specifically apply to premenopausal women; therefore, women over the age of 50 years were excluded to minimize the number of postmenopausal women. Because the IDSA guidelines apply only to uncomplicated UTIs, patients with *ICD-10* codes for the following conditions were excluded: current pregnancy, history of pyelonephritis, history of diabetes, history of any organ transplant, history of human immunodeficiency virus, immunosuppression, renal insufficiency, urinary tract abnormalities, and history of urologic procedures.

Characteristics of the treating clinician were extracted and included clinician type (physician, physician assistant, nurse practitioner, and other), specialty, and care setting (direct or private sector care). Patient characteristics including age, race (American Indian and Alaskan Native, Asian or Pacific Islander, Black, White, Other), beneficiary status (active duty, military dependent, retiree, other), and military rank of the patient or sponsor (junior enlisted, senior enlisted, junior officer, senior officer, warrant officer, and other) were also extracted. This study retained MDR-reported categories of race, with the exception of unknown and missing race categorized together by the authors and reported as missing race. The other race category was a self-selected value available to MHS beneficiaries when reporting their race. Race in the MDR does not include ethnicity; therefore reported categories are of race only.

### Statistical Analysis

Study analyses included descriptive statistics of patient and clinician characteristics, IDSA guideline concordance rates by patient and clinician characteristics, and both unadjusted and adjusted logistic regressions with 95% CIs for the receipt of IDSA guideline-concordant treatment. Frequency comparisons of clinician specialties in direct vs private sector care were added as follow-up analyses. The IDSA guideline-concordance rates among women with uncomplicated UTIs were calculated as the number of cases receiving first-line guideline antibiotic therapy divided by the total number of cases for uncomplicated UTI. All patient and clinician characteristics were used as factors in all logistic regression modeling, and patients with missing values for variables were not included in regression analyses. A high rate of overprescribing was observed in women receiving nonconcordant treatment; therefore, additional regression modeling was performed on data from the subset of women receiving nonconcordant treatment. Because race was missing for nearly 34% of the study population and was a desired assessment as a factor in both guideline-concordant treatment and overprescribing, the reweighted estimating equations method^22^ with patient age, beneficiary status, and rank used as variables for the observed probabilities, was used in the adjusted regression models. Complete case analysis models with 95% CIs were used for sensitivity testing of the reweighted estimating equations regression results. All analyses were set at a priori probability of α < .05. All analyses were performed using SAS software, version 9.4 (SAS Institute Inc).

## Results

Between October 1, 2017, and September 30, 2019, there were 46 793 US women diagnosed with an uncomplicated UTI. Detailed patient demographic characteristics and clinical characteristics are presented in Table 1. Most patients were aged 18 to 34 years (67.3% [31 475 of 46 793]), of White race (38.2% [17 859 of 46 793]), and most had 1 UTI during the study period (85.9% [40 225 of 46 793]). Additional clinical characteristics associated with the first UTI during the study period are presented in Table 2. Most women received IDSA guideline-concordant treatment (91.0% [42 583 of 46 793]) and received care in the direct care setting (56.8% [26 580 of 46 793]). The IDSA guideline concordance rate was 92.5% in direct care and 89.0% in private sector care. Differences in UTI diagnosis by specialties and care settings were also examined (eTable 2 in the Supplement). The specialties with the highest diagnostic rates in direct care were clinicians with unknown specialties (34.2%), primary care (23.3%), and emergency medicine (18.9%). In private sector care, the specialties with the highest diagnostic rates were clinicians with other specialties (32.9%), family medicine (25.3%), and clinicians with unknown specialties (14.9%).

**Table 1.** Patient Demographic and Clinical Characteristics

Variable	No. (%)
Total No.	46 793
Age group, y	
18-24	16 432 (35.1)
25-34	15 043 (32.2)
35-44	9955 (21.3)
45-50	5363 (11.5)
Race^a^	
American Indian or Alaska Native	263 (0.6)
Asian or Pacific Islander	1691 (3.6)
Black	5989 (12.8)
White	17 859 (38.2)
Missing	15 868 (33.9)
Other	5123 (10.9)
Beneficiary status	
Dependent	34 718 (74.2)
Active duty	10 676 (22.8)
Other	138 (0.3)
Missing	30 (0.1)
Retiree	1231 (2.6)
Branch	
Army	19 612 (41.9)
Air Force	12 938 (27.7)
Navy	9536 (20.4)
Marine Corps	4707 (10.1)
Rank	
Junior enlisted	11 527 (24.6)
Senior enlisted	26 859 (57.4)
Junior officer	4664 (9.9)
Senior officer	2417 (5.2)
Warrant officer	1182 (2.5)
Other	144 (0.3)
No. of UTIs during study period	
1	40 225 (85.9)
2	6242 (13.3)
3 or more	326 (0.7)

Abbreviation: UTIs, urinary tract infections.

^a^ Race in the Military Health System Data Repository does not include ethnicity; therefore reported categories are of race only. Other race is a reported value in the Military Health System Data Repository and is self-selected by Military Health System beneficiaries. Beneficiaries could select it to identify as multiracial, however details of race combinations are not reported or available.

**Table 2.** Clinical and Clinician Characteristics and Guideline-Concordant Treatment

Variable	No. (%)
Care setting	
Direct care	26 580 (56.8)
Private sector care	20 213 (43.2)
Guideline-concordant treatment	
Yes	42 583 (91.0)
No	4210 (9.0)
Over prescribing	2770 (5.9)
Clinician type	
Other	11 216 (23.9)
Physician assistant/nurse practitioner	14 721 (31.5)
Physician	20 856 (44.6)
Clinician specialty	
Clinician, unknown specialty	12 102 (25.9)
Family medicine	8657 (18.5)
Emergency medicine	8019 (17.1)
Other	8016 (17.1)
Primary care	6750 (14.4)
Internal medicine	1479 (3.2)
Obstetrics and gynecology	1333 (2.9)
Pediatrics	177 (0.4)
Urology	148 (0.3)
Surgery	112 (0.2)

Table 3 details the unadjusted and adjusted reweighted estimating equations logistic regression results for receipt of IDSA guideline-concordant treatment, and all logistic regression models are presented in eTable 3 in the Supplement. In comparison of the unadjusted with adjusted results, changes in significance were observed but no substantial changes were observed in the odds ratios (OR). In comparison with the reference group of age 25 to 34 years, women in all other age groups were less likely to receive IDSA guideline-concordant treatment. Asian or Pacific Islander (aOR, 1.26; 95% CI, 1.23-1.28) and Black (aOR, 1.04; 95% CI, 1.03-1.05) race were more likely to receive IDSA guideline-concordant treatment in comparison with White women. Compared with obstetrics and gynecology, urology had lower rates of guideline-concordant treatment (adjusted OR [aOR], 0.40; 95% CI, 0.38-0.43) and all the other specialties had higher rates of IDSA guideline concordance: internal medicine (aOR, 2.87; 95% CI, 2.73-3.03), family medicine (aOR, 1.81; 95% CI, 1.76-1.87), surgery (aOR, 1.51; 95% CI, 1.36-1.67), and emergency medicine (aOR, 1.36; 95% CI, 1.32-1.39). Only 3.4% of UTI treatments were within a surgical specialty. Private sector care had lower rates of IDSA guideline concordance (aOR, 0.63; 95% CI, 0.62-0.64) compared with direct care.

**Table 3.** Logistic Regression Results for Guideline-Concordant Treatment for Uncomplicated UTIs

Variable	OR (95% CI)	
	Unadjusted	Adjusted reweighted estimating equations (n = 30 902)
Age group, y		
18-24	0.98 (0.89-1.08)	0.89 (0.88-0.91)^a^
25-34	1 [Reference]	1 [Reference]
35-44	0.89 (0.80-0.99)^a^	0.75 (0.74-0.76)^a^
45-50	0.88 (0.77-1.01)	0.91 (0.88-0.93)^a^
Race^b^		
American Indian or Alaska Native	0.79 (0.53-1.16)	0.92 (0.88-0.96)^a^
Asian or Pacific Islander	1.16 (0.96-1.39)	1.26 (1.23-1.28)^a^
Black	1.00 (0.90-1.11)	1.04 (1.03-1.05)^a^
White	1 [Reference]	1 [Reference]
Other	0.98 (0.88-1.09)	0.94 (0.93-0.96)^a^
Beneficiary status		
Active duty	1.14 (1.04-1.24)^a^	1.07 (1.04-1.11)^a^
Dependents	1 [Reference]	1 [Reference]
Other	0.95 (0.51-1.78)	0.66 (0.53-0.82)^a^
Retiree	0.87 (0.72-1.06)	0.94 (0.90-0.98)^a^
Rank		
Other	1.39 (0.56-3.45)	1.20 (0.70-2.04)
Junior enlisted	1 [Reference]	1 [Reference]
Senior enlisted	0.97 (0.89-1.07)	1.12 (1.10-1.13)^a^
Junior officer	1.04 (0.90-1.21)	1.01 (0.99-1.03)
Senior officer	1.12 (0.91-1.38)	1.14 (1.10-1.18)^a^
Warrant officer	1.04 (0.80-1.35)	2.33 (2.20-2.47)^a^
Clinician type		
Physician	1 [Reference]	1 [Reference]
Other	0.92 (0.84-1.01)	0.94 (0.91-0.96)^a^
Physician assistant/nurse practitioner	1.24 (1.13-1.36)^a^	0.97 (0.96-0.99)
Clinician specialty		
Obstetrics and gynecology	1 [Reference]	1 [Reference]
Emergency medicine	1.48 (1.19-1.84)^a^	1.36 (1.32-1.39)^a^
Family medicine	1.59 (1.27-1.98)^a^	1.81 (1.76-1.87)^a^
Internal medicine	1.55 (1.13-2.12)^a^	2.87 (2.73-3.03)^a^
Other	1.26 (1.02-1.57)^a^	1.55 (1.50-1.60)^a^
Primary care	2.02 (1.62-2.53)^a^	1.54 (1.51-1.58)^a^
Clinician, unknown specialty	1.74 (1.41-2.15)^a^	1.60 (1.56-1.64)^a^
Surgery	1.02 (0.49-2.11)	1.51 (1.36-1.67)^a^
Urology	0.60 (0.35-1.03)	0.40 (0.38-0.43)^a^
Care setting		
Direct care	1 [Reference]	1 [Reference]
Private sector	0.65 (0.60-0.70)^a^	0.63 (0.62-0.64)^a^

Abbreviation: OR, odds ratio.

^a^ Indicates statistical significance with *P* = .05.

^b^ Race in the Military Health System Data Repository does not include ethnicity; therefore reported categories are of race only. Other race is a reported value in the Military Health System Data Repository and is self-selected by Military Health System beneficiaries. Beneficiaries could select it to identify as multiracial, however details of race combinations are not reported or available.

Of the 4210 women who did not receive IDSA guideline-concordant treatment (9.0% of the study population), the antibiotic overtreatment rate was 5.9% (Table 4). Compared with obstetrics and gynecology, there were higher overprescribing rates seen in emergency medicine (aOR, 3.78; 95% CI, 3.55-4.01) and family medicine (aOR, 3.50; 95% CI, 3.28-3.74) (eTable 4 in the Supplement). Compared with direct care, there was a lower overprescribing rate in private sector care (aOR, 0.54; 95% CI, 0.53-0.56).

**Table 4.** Unadjusted and Adjusted Regression Results for the Probability of Overprescribing Antibiotics

Variable	OR (95% CI)	
	Unadjusted	Adjusted reweighted estimating equations (n = 2763)
Age group, y		
18-24	0.94 (0.78-1.15)	0.92 (0.89-0.95)^a^
25-34	1 [Reference]	1 [Reference]
35-44	1.24 (1.00-1.54)^a^	0.65 (0.63-0.67)^a^
45-50	1.35 (1.03-1.78)^a^	1.05 (0.97-1.13)
Race^b^		
American Indian or Alaska Native	0.76 (0.36-1.61)	0.73 (0.66-0.80)^a^
Asian or Pacific Islander	0.72 (0.50-1.03)	0.77 (0.73-0.81)^a^
Black	0.79 (0.64-0.97)^a^	0.74 (0.72-0.76)^a^
White	1 [Reference]	1 [Reference]
Other	0.91 (0.73-1.14)	1.07 (1.03-1.12)^a^
Beneficiary status		
Active duty	0.86 (0.73-1.02)	0.80 (0.74-0.86)^a^
Dependents	1 [Reference]	1 [Reference]
Other	2.19 (0.47-10.18)	1.98 (0.99-3.95)
Retiree	1.45 (0.96-2.19)	1.84 (1.68-2.02)^a^
Rank		
Junior enlisted	1 [Reference]	1 [Reference]
Senior enlisted	1.27 (1.06-1.52)^a^	1.19 (1.15-1.23)^a^
Junior officer	1.48 (1.09-2.02)^a^	1.44 (1.38-1.51)^a^
Senior officer	1.26 (0.83-1.91)	1.36 (1.27-1.45)^a^
Warrant officer	1.35 (0.79-2.31)	4.93 (3.99-6.09)^a^
Other	0.40 (0.07-2.43)	0.28 (0.13-0.57)^a^
Clinician type		
Physician	1 [Reference]	1 [Reference]
Physician assistant/nurse practitioner	1.23 (1.01-1.49)^a^	1.62 (1.55-1.70)^a^
Other	0.63 (0.52-0.76)^a^	0.94 (0.89-0.99)^a^
Clinician specialty		
Obstetrics and gynecology	1 [Reference]	1 [Reference]
Emergency medicine	1.82 (1.20-2.77)^a^	3.78 (3.55-4.01)^a^
Family medicine	1.93 (1.26-2.97)^a^	3.50 (3.28-3.74)^a^
Internal medicine	1.50 (0.80-2.83)	0.94 (0.82-1.07)
Other	1.04 (0.68-1.57)	2.75 (2.56-2.95)^a^
Primary care	1.54 (1.00-2.37)	2.57 (2.42-2.72)^a^
Clinician, unknown specialty	1.60 (1.07-2.40)^a^	1.99 (1.87-2.11)^a^
Surgery	2.63 (0.52-13.20)	0.57 (0.44-0.73)^a^
Urology	0.40 (0.15-1.09)	4.22 (3.67-4.85)^a^
Care setting		
Direct care	1 [Reference]	1 [Reference]
Private sector	0.76 (0.65-0.89)^a^	0.54 (0.53-0.56)^a^

Abbreviation: OR, odds ratio.

^a^ Indicates statistical significance with *P* = .05.

^b^ Other race is a reported value in the Military Health System Data Repository and is self-selected by Military Health System beneficiaries. Beneficiaries could select it to identify as multiracial, however details of race combinations are not reported or available.

## Discussion

The overall IDSA guideline-concordance rate for uncomplicated UTIs among adult women between 18 to 50 years of age within the MHS is high at 91.0%, with higher rates observed in direct care compared with private sector care. Despite this high IDSA guideline-concordance rate, there is variability among specialties with lower IDSA guideline-concordance rates observed in urology and obstetrics and gynecology compared with other specialties. Of the 9% discordance rate, the antibiotic overtreatment rate was 5.9%, with higher rates of overtreatment in emergency medicine and family medicine.

The MHS IDSA guideline-concordance rate of 91% encompasses both direct care and private sector care and is substantially higher than what has been previously reported in the literature.^9^ Even the MHS IDSA guideline-concordance rate among private sector care was substantially higher than previous studies at 89.0%. A prior study reported IDSA guideline-concordance rates of up to 64%, but this study included women up to 75 years of age and was a retrospective observational secondary analysis of the National Disease and Therapeutic Index, which used a sample of 4800 office-based physicians to calculate national projections.^9^ Our study used health care data directly from the MDR, and we used strict exclusion criteria to best match the patient population described in the IDSA guidelines, which may have been a factor in the higher IDSA guideline-concordance rates. In addition, the IDSA clinical practice guidelines were published in 2011, a time frame that has given clinicians at least 7 years to learn and implement the IDSA guidelines. It is less clear why the IDSA guideline-concordance rate was substantially higher in direct care. One factor could be that the military health system uses 1 universal medical record system, whereas the electronic medical record may be more fragmented among various facilities in the private sector. Despite a universally insured patient population, the concordance rates were higher in direct care, suggesting that there may be underlying differences with prescribing choices between these 2 care settings. It raises the question as to whether universal health care may be associated with greater adherence to clinical practice guidelines.

The rate of antibiotic overtreatment was 5.9% with higher rates in emergency medicine, family medicine, and other. Although obstetrics and gynecology and urology had lower overall rates of IDSA guideline concordance, they also had lower rates of antibiotic overtreatment in comparison with other specialties. In comparison with private sector care, rates of antibiotic overtreatment were higher in direct care facilities. It has previously been speculated that better insurance coverage may be associated with more expensive overtreatment for UTIs owing to fewer restrictions on prescribing choices.^9^ Another factor associated with antibiotic overtreatment seen in direct care may be that military clinicians may not encounter as many barriers regarding insurance coverage for antibiotic prescriptions as frequently as private sector clinicians, so they may be more prone to overtreatment. Although overtreatment rates were higher in direct care compared with private sector care, the overall IDSA guideline-concordance rate was substantially higher in direct care.

It has previously been shown that clinicians in obstetrics and gynecology and urology have higher rates of IDSA guideline concordance^9^ and emergency medicine has lower rates^23,24^; however, the results of this study are not consistent with those findings. Clinicians in internal medicine, primary care, and emergency medicine had higher IDSA guideline-concordance rates and urology had lower IDSA guideline-concordance rates. Of note, all the surgical specialties combined accounted for only 3.4% of UTI treatments within the MHS, which may indicate that surgical specialties do not manage uncomplicated UTIs as frequently as general medicine specialties. It was particularly surprising that urology had lower IDSA concordance rates in our study, which is not concordant with a prior study.^9^

There are conflicting reports^9,13^ regarding racial disparities for the treatment of uncomplicated UTIs. Our study found higher IDSA guideline-concordance rates among Asian and Black women and lower rates in Native American/Alaska Native women and other. This finding conflicts with a prior study, in which Asian and White women were found to have lower rates of IDSA guideline concordance.^9^ Previous studies on the MHS have reported the mitigation of many, but not all, racial disparities in health care for the MHS population.^25^ There were also lower rates of IDSA guideline concordance in the lower (18 to 24 years) and upper (35 to 50 years) age ranges. The age cutoff of 50 years was used to exclude postmenopausal women. Given that the average age of menopause in the US is 51, the age group of 45 to 50 years likely includes some menopausal patients, which could affect concordance rates. In addition, older age is a risk factor associated with antibiotic allergies and multidrug resistance.^26,27^ These findings on older age and guideline concordance are consistent with a prior study.^9^ It is unclear why the concordance rates are lower in the age group of 18 to 24 years whereas another study found higher concordance rates in this age group.^9^

### Limitations

This study has limitations. Allergy information was not available, which is an important factor when addressing antibiotic prescriptions. Reported rates of antibiotic allergies range from 4.4% to 4.9%,^27,28^ which is lower than the 9% discordance rate observed in our study. For guideline concordance, we did not specifically analyze the dosage or duration of the antibiotic therapy. In addition, urine culture results were not available, and we limited antibiotic prescriptions to within 24 hours of the diagnosis to minimize any potential antibiotic adjustments following urine culture results. It was also not possible to determine which patients received antibiotic therapy for asymptomatic bacteriuria. In addition, secondary health care claims data was used and is subject to coding error.

## Conclusions

To our knowledge, this is the first study assessing IDSA guideline concordance for uncomplicated UTIs in the US Military Health System. Our study encompasses a wide breadth of universally insured patients receiving care in both direct and private sector facilities and captures clinical practice patterns across various specialties and clinician types. The results of our study further enhance the literature on current antibiotic prescribing practices for uncomplicated UTIs in adult women. Lower rates of IDSA guideline concordance were seen in obstetrics and gynecology and urology, which could potentially benefit from targeted antibiotic stewardship programs and policies that promote greater adherence to IDSA guidelines. Further research incorporating antibiotic allergy information, antibiotic resistance, and antibiotic prescribing patterns in relation to urine cultures is warranted to better understand the complexities of current antibiotic prescribing practices and optimize the treatment of UTIs.

## Notes

Supplement. **eTable 1.** List of IDSA Concordant and Non-Concordant Antibiotics **eTable 2.** Provider Specialty by Care Setting **eTable 3.** All Logistic Regression Results for Probability of IDSA Concordant Treatment **eTable 4.** All Regression Results for the Probability of Over Prescribing Antibiotics
