# Supplementary material for: Antibiotic Prescribing Patterns and Guideline Concordance for Uncomplicated Urinary Tract Infections Among Adult Women in the US Military Health System
Source: JAMA Netw Open. 2022 Aug 4;5(8):e2225730. doi: 10.1001/jamanetworkopen.2022.25730 (PMC9353594; doi:10.1001/jamanetworkopen.2022.25730)
Supplement: Supplement. — eTable 1. List of IDSA Concordant and Non-Concordant Antibiotics eTable 2. Provider Specialty by Care Setting eTable 3. All Logistic Regression Results for Probability of IDSA Concordant Treatment eTable 4. All Regression Results for the Probability of Over Prescribing Antibiotics [file jamanetwopen-e2225730-s001.pdf]

Supplemental Online Content

Kikuchi JY, Banaag A, Koehlmoos TP. Antibiotic prescribing patterns and guideline concordance for uncomplicated urinary tract infections among adult women in the US military health system. *JAMA Netw Open.* 2022;5(8):e2225730. doi:10.1001/jamanetworkopen.2022.25730

eTable 1. List of IDSA Concordant and Non-Concordant Antibiotics

eTable 2. Provider Specialty by Care Setting

eTable 3. All Logistic Regression Results for Probability of IDSA Concordant Treatment

eTable 4. All Regression Results for the Probability of Over Prescribing Antibiotics

This supplemental material has been provided by the authors to give readers additional information about their work.

© 2022 Kikuchi JY et al. *JAMA Network Open.*

eTable 1. List of IDSA concordant and non-concordant antibiotics

**IDSA Concordant Antibiotics**
Nitrofurantoin
Furadantin
Macrobid
Macrodantin
Trimethoprim
Bactrim
Sulfatrim
Fosfomycin
Monurol
Pivmecillinam
**IDSA Non-concordant Antibiotics**
Ofloxacin
Floxin
Ciprofloxacin
Cipro
Gemifloxacin
Factive
Levofloxacin
Levaquin
Amoxicillin
Augmentin
Cefdinir
Omnicef
Cefaclor
Ceclor
Raniclor
Cefpodoxime
Vantin
Cefadroxil
Duricef
Cephalexin
Daxbia
Keflex
Amoxil
Moxatag
Trimox
Ampicillin
Ampi
Omnipen
Penglobe
Principen
Ertapenem

© 2022 Kikuchi JY et al. *JAMA Network Open.*

Invanz
Ceftriaxone
Gentamicin
Tobramycin

© 2022 Kikuchi JY et al. *JAMA Network Open.*

eTable 2. Provider specialty by care setting

Provider Specialty	Total (n=46,793)	Direct Care (n=26,580)	Private Sector Care (n=20,213)
		n (row %)	
Provider, unknown specialty	12102	9084 (75.06)	3018 (24.94)
Family Medicine	8657	3551 (41.02)	5106 (58.98)
Emergency Medicine	8019	5036 (62.80)	2983 (37.20)
Other	8193	1352 (16.50)	6664 (83.50)
Primary Care	6750	6198 (91.82)	552 (8.18)
Internal Medicine	1479	395 (26.71)	1084 (73.29)
OBGYN	1333	828 (62.12)	505 (37.88)
Urology	148	70 (47.30)	107 (52.70)
Surgery	112	28 (25.00)	120 (75.00)

© 2022 Kikuchi JY et al. *JAMA Network Open.*

eTable 3. All logistic regression results for probability of IDSA concordant treatment

	Unadjusted OR (95% CI)	Adjusted CCA OR (95% CI) (n=30,902)	Adjusted RWEE OR (95% CI) (n=30,902)
**Age Group**			
18 to 24	0.98 (0.89 - 1.08)	0.98 (0.88 - 1.10)	0.89 (0.88-0.91)*
25 to 34 (reference)	1	1	1
35 to 44	0.89 (0.80 - 0.99)*	0.89 (0.80 - 1.00)*	0.75 (0.74-0.76)*
45 to 50	0.88 (0.77 - 1.01)	0.88 (0.77 - 1.02)	0.91 (0.88 - 0.93)*
**Race**			
American Indian/Alaska Native	0.79 (0.53 - 1.16)	0.80 (0.54 - 1.18)	0.92 (0.88 - 0.96)*
Asian/Pacific Islander	1.16 (0.96 - 1.39)	1.12 (0.93 - 1.34)	1.26 (1.23 - 1.28)*
Black	1.00 (0.90 - 1.11)	0.99 (0.89 - 1.10)	1.04 (1.03 - 1.05)*
Other	0.98 (0.88 - 1.09)	0.94 (0.84 - 1.05)	0.94 (0.93 - 0.96)*
White (reference)	1	1	1
**Beneficiary Status**			
Active Duty	1.14 (1.04 - 1.24)*	1.05 (0.95 - 1.16)	1.07 (1.04 - 1.11)*
Dependents (reference)	1	1	1
Other	0.95 (0.51 - 1.78)	0.83 (0.44 - 1.55)	0.66 (0.53 - 0.82)*
Retiree	0.87 (0.72 - 1.06)	0.94 (0.77 - 1.15)	0.94 (0.90 - 0.98)*
**Rank**			
Other	1.39 (0.56 - 3.45)	1.18 (0.47 - 2.93)	1.20 (0.70 - 2.04)
Junior Enlisted (reference)	1	1	1
Senior Enlisted	0.97 (0.89 - 1.07)	1.11 (0.98 - 1.25)	1.12 (1.10 - 1.13)*
Junior Officer	1.04 (0.90 - 1.21)	1.17 (0.99 - 1.38)	1.01 (0.99 - 1.03)
Senior Officer	1.12 (0.91 - 1.38)	1.28 (1.02 - 1.61)*	1.14 (1.10 - 1.18)*
Warrant Officer	1.04 (0.80 - 1.35)	1.21 (0.92 - 1.60)	2.33 (2.20 - 2.47)*
**Provider Type**			
Physician (reference)	1	1	1
Other	0.92 (0.84 - 1.01)	0.87 (0.70 - 1.09)	0.94 (0.91 - 0.96)*
PA/NP	1.24 (1.13 - 1.36)*	1.10 (0.93 - 1.30)	0.97 (0.96 - 0.99)
**Provider Specialty**			
OBGYN (reference)	1	1	1
Emergency Medicine	1.48 (1.19 - 1.84)*	1.60 (1.27-2.02)*	1.36 (1.32 - 1.39)*
Family Medicine	1.59 (1.27 - 1.98)*	1.84 (1.46-2.33)*	1.81 (1.76 - 1.87)*
Internal Medicine	1.55 (1.13 - 2.12)*	1.94 (1.40-2.68)*	2.87 (2.73 - 3.03)*
Other	1.26 (1.02-1.57)*	1.78 (1.35-2.35)*	1.55 (1.50 - 1.60)*
Primary Care	2.02 (1.62 - 2.53)*	1.82 (1.45-2.29)*	1.54 (1.51 - 1.58)*

© 2022 Kikuchi JY et al. *JAMA Network Open.*

Provider, unknown specialty	1.74 (1.41 - 2.15)*	1.70 (1.34-2.15)*	1.60 (1.56 - 1.64)*
Surgery	1.02 (0.49 - 2.11)	1.19 (0.57-2.47)	1.51 (1.36 - 1.67)*
Urology	0.60 (0.35 - 1.03)	0.79 (0.45-1.36)	0.40 (0.38 - 0.43)*
**Care Setting**			
Direct Care (reference)	1	1	1
Private Sector	0.65 (0.60 - 0.70)*	0.66 (0.60 - 0.73)*	0.63 (0.62 - 0.64)*

CCA Complete Care Analysis

RWEE Reweighted Estimating Equations

OR Odds Ratio

CI Confidence Interval

© 2022 Kikuchi JY et al. *JAMA Network Open.*

eTable 4. All regression results for the probability of over prescribing antibiotics

	Unadjusted OR (95% CI)	Adjusted CCA OR (95% CI) (n=2,763)	Adjusted RWEE OR (95% CI) (n=2,763)
**Age Group**			
18 to 24	0.94 (0.78 - 1.15)	1.00 (0.80 - 1.26)	0.92 (0.89 - 0.95)*
25 to 34 (ref)	1	1	1
35 to 44	1.24 (1.00 - 1.54)*	1.23 (0.98 - 1.54)	0.65 (0.63 - 0.67)*
45 to 50	1.35 (1.03 - 1.78)*	1.27 (0.94 - 1.71)	1.05 (0.97 - 1.13)
**Race**			
American Indian/Alaska Native	0.76 (0.36 - 1.61)	0.73 (0.33 - 1.58)	0.73 (0.66 - 0.80)*
Asian/Pacific Islander	0.72 (0.50 - 1.03)	0.66 (0.45 - 0.96)*	0.77 (0.73 - 0.81)*
Black	0.79 (0.64 - 0.97)*	0.72 (0.58 - 0.89)*	0.74 (0.72 - 0.76)*
Other	0.91 (0.73 - 1.14)	0.88 (0.70 - 1.11)	1.07 (1.03 - 1.12)*
White (ref)	1	1	1
**Beneficiary Status**			
Active Duty	0.86 (0.73 - 1.02)	0.96 (0.78 - 1.19)	0.80 (0.74 - 0.86)*
Dependents (ref)	1	1	1
Other	2.19 (0.47 - 10.18)	2.30 (0.48 - 11.09)	1.98 (0.99 - 3.95)
Retiree	1.45 (0.96 - 2.19)	1.55 (1.00 - 2.40)*	1.84 (1.68 - 2.02)*
**Rank**			
Junior Enlisted (ref)	1	1	1
Senior Enlisted	1.27 (1.06 - 1.52)*	1.20 (0.94 - 1.54)	1.19 (1.15 - 1.23)*
Junior Officer	1.48 (1.09 - 2.02)*	1.45 (1.02 - 2.06)*	1.44 (1.38 - 1.51)*
Senior Officer	1.26 (0.83 - 1.91)	1.01 (0.63 - 1.61)	1.36 (1.27 - 1.45)*
Warrant Officer	1.35 (0.79 - 2.31)	1.21 (0.68 - 2.18)	4.93 (3.99 - 6.09)*
Other	0.40 (0.07 - 2.43)	0.36 (0.06 - 2.22)	0.28 (0.13 - 0.57)*
**Provider Type**			
Physician (ref)	1	1	1
PA/NP	1.23 (1.01 - 1.49)*	1.65 (1.15 - 2.38)*	1.62 (1.55 - 1.70)*
Other	0.63 (0.52 - 0.76)*	0.73 (0.47 - 1.16)	0.94 (0.89 - 0.99)*
**Provider Specialty**			
OBGYN (ref)	1	1	1
Emergency Medicine	1.82 (1.20 - 2.77)*	2.26 (1.44-3.55)*	3.78 (3.55 - 4.01)*
Family Medicine	1.93 (1.26 - 2.97)*	2.26 (1.42-3.58)*	3.50 (3.28 - 3.74)*
Internal Medicine	1.50 (0.80 - 2.83)	1.90 (0.98-3.70)*	0.94 (0.82 - 1.07)
Other	1.04 (0.68-1.57)	1.95 (1.13-3.36)*	2.75 (2.56 - 2.95)*
Primary Care	1.54 (1.00 - 2.37)	1.22 (0.77-1.93)	2.57 (2.42 - 2.72)*
Provider, unknown specialty	1.60 (1.07 - 2.40)*	1.59 (0.99-2.55)	1.99 (1.87 - 2.11)*

© 2022 Kikuchi JY et al. *JAMA Network Open.*

Surgery	2.63 (0.52 - 13.20)	2.94 (0.57-15.10)	0.57 (0.44 - 0.73)*
Urology	0.40 (0.15 - 1.09)	0.47 (0.17-1.30)	4.22 (3.67 - 4.85)*
**Care Setting**			
Direct Care (ref)	1	1	1
Private Sector	0.76 (0.65 - 0.89)*	0.72 (0.59 - 0.88)*	0.54 (0.53 - 0.56)*

© 2022 Kikuchi JY et al. *JAMA Network Open.*
